# Multi-detector row computed tomography (MDCT) and magnetic resonance imaging (MRI) in the evaluation of the mandibular invasion by squamous cell carcinomas (SCC) of the oral cavity. Correlation with pathological data

**DOI:** 10.1186/1756-9966-29-73

**Published:** 2010-06-17

**Authors:** Antonello Vidiri, Antonino Guerrisi, Raul Pellini, Valentina Manciocco, Renato Covello, Oreste Mattioni, Isabella Guerrisi, Salvatore Di Giovanni, Giuseppe Spriano, Marcello Crecco

**Affiliations:** 1 Department of Radiology and Diagnostic Imaging, Regina Elena Institute, E. Chianesi 53, Rome, Italy; 2 Department of Radiological Sciences, University of Rome Sapienza, Viale Regina 324 Rome Italy; 3 Department of Otholaringology and Maxillo-Facial Surgery, Regina Elena Institute, E.Chianesi 53, Rome, Italy; 4 Department of Pathology, Regina Elena Institute, E.Chianesi 53, Rome, Italy; 5 Department of Bioimages and Radiological Sciences "A. Gemelli" University Hospital, Largo Francesco Vito 1, Rome, Italy

## Abstract

**Background:**

To retrospectively compare the diagnostic accuracy of magnetic resonance imaging (MRI) and multidetector-row computed tomography (MDCT) in the assessment of the mandibular invasion by squamous cell carcinoma (SCC) having histopathological exams as standard of reference.

**Materials and methods:**

Institutional review board approval with a waiver of informed patient consent was obtained. Of the 147 patients selected from our database who underwent surgical excision of a tumour arising into the oral cavity, thirty-six patients (26 men, 10 women; mean age, 56 years; range, 30-75 years) with hystologically proven SCC who performed both a preoperative MRI and MDCT, composed our final study population.

Images were qualitatively analyzed in consensus by two expert radiologist in head and neck imaging. Sensitivity, specificity, accuracy, positive predictive value (PPV) and negative predictive value (NPV) were assessed for both MRI and MDCT.

Differences in sensitivity, specificity, positive and negative predictive values were calculated at a statistical significance of p < .05.

**Results:**

The sensitivity, the specificity and the accuracy of MRI and MDCT in the detection of the mandibular involvement were respectively 93%, 82%, 86% and 79%, 82%, 81%, while the positive predictive value (PPV) and negative predictive value (NPV) were respectively 76%, 95% and 73%, 86%. There wasn't any statistically significant difference in overall diagnostic accuracy between MRI and MDCT in the evaluation of mandibular tumour invasion (p > .05).

**Conclusion:**

MRI showed to have a higher sensitivity compare to MDCT in the assessment of mandibular involvement from SCC arising in the oral cavity although none statistically significant differences were noted.

## Background

An adequate staging of a tumour arising in the oral-cavity is essential for the choice of appropriate surgical management (i.e. ablative, reconstructive) and for the chemo-radiation therapy planning [1,2]. The evaluation of either the depth or the extension of the invasion of both the soft tissue and the bone adjacent to the lesion is necessary to well stage the oral-cavity tumours. This is particularly emphasized when a mandibular involvement is presumable, considering the probable tumour invasion of both its cortical and medullary components.

Clinical assessment of mandibular invasion is possible by either evaluating clinical symptoms and signs or bimanually assessing the mobility of the tumour in relation to the mandible [3]. However, the clinical examination always requires an imaging correlation. Various imaging techniques (i.e. ortopanthomography, scintigraphy, computed tomography, magnetic resonance imaging, positron emission tomography) are actually used to make a diagnosis of mandibular invasion by tumours of the oral cavity [4-6].

Multidetector-row computed tomography (MDCT) and Magnetic Resonance Imaging (MRI) represent the routine imaging modalities for the pre-operative tumour staging of oral and oropharyngeal squamous cell carcinoma (SCC). These techniques provide multiple informations regarding (i) the extension of the tumour beyond the midline lingual septum, (ii) the deep extension and/or (iii) the infiltration of the mandible, considering either the cortical or medullary portion [7-9], all of them considered very important points for treatment planning [10-12]. However, in some cases also with imaging it could be difficult to determine exactly the presence and rate of bone infiltration, and particularly to establish the involvement of the cortical and/or medullary part of the mandible [3,12-14]. To our knowledge very few studies compared MDCT and MRI in the evaluation of the mandibular involvement from tumours arising into the oral cavity. Moreover, whereas these authors reported often different results [4,7,9,12], which of these modalities has a better diagnostic accuracy remains still controversial. The more plausible explanation to these different results could be due to the fact that most of these studies were not comparable, because of the different study methods or study design adopted. However, despite these studies varied widely, at our careful review of the literature data, MRI is resulted superior to MDCT in the evaluation of the medullary involvement while MDCT is resulted more accurate compare to MRI in the visualization of small cortical bone erosions [4,7,9].

The aim of this study was to assess the accuracy of both MRI and MDCT and to compare these imaging techniques in the evaluation of the mandibular tumour invasion; successively we correlated the results of the radiological analysis with the histopathological results that represented our reference standard.

## Methods

This retrospective study was approved by the local institutional review committee, with a waiver of written informed consent.

### Patients Population

147 patients who underwent surgical procedures between january 2003 and december 2007 for excision of a tumour arising into the oral cavity were retrospectively selected from our database. All patients enrolled in the final study population had to satisfy the following inclusion criteria: (i) both surgical procedure and preoperative imaging examinations performed in our istitution, (ii) a clinical evaluation of the mandibular infiltration, (iii) having the results of histophatological examinations. Exclusion criteria were the following: (i) patients who performed only MDCT (n = 4) or only MRI (n = 37) examinations; (ii) lack of histopathological confirmation of SCC (n = 19); (iii) preoperative treatments with radiotherapy and/or chemotherapy (n = 24); (iv) a time greater than two weeks between the two examination (n = 20); (v) the presence of metallic artifacts in the images that could interfere with radiological interpretation (n = 7). Thirty-six patients (26 men and 10 women) composed our final study population (table 1). A chart review of clinical and pathological data was conducted by a surgeon (R.P.) and by a pathologist (R.C.) in order to recover either clinical or pathological data.

**Table 1:** Demographic and clinical findings of the study patients (N = 36)

**Age (years) - mean (range)**	56 (30-75)
	
**Gender - no. (%)**	
Male	26 (72)
Female	10 (28)
	
**Weight (kg) - mean (range)**	72 (52-85)
	
**Body mass index (kg/m^2^) - mean (range)**	22 (19-27)
	
**Race or ethnic group - no. (%)**	
White	35 (97)
Black	0
Other	1 (3)
	
**Time interval between MDCT and MRI examinations (days)**	
Mean	9
Range	4-14
	
**Clinical Stadiation (T) - no. (%)**	
T4	21 (58)
T3	5 (14)
T2	6 (17)
T1	4 (11)
	
**Type of surgical procedure performed - no. (%)**	
Commando procedure	9 (25)
Segmental resection with fibula	15 (42)
Marginal resection	12 (33)
	

Note. Percentages may not total 100 because of rounding.

Tumours ranged in size from 4 to 32 mm and arised from the floor of mouth (n = 18), gingiva (n = 10), retromolar trigone (n = 4 ), alveolar ridge (n = 3) and lip (n = 1).

### Imaging Techniques

MRI was obtained by the use of a 0.5 T superconductive system (Gyroscan, Philips healthcare , Eindhoven, The Netherlands). MRI was performed using a neck-coil, 5-millimeter-thick slice, two acquisitions and a matrix of 256 × 256 pixels. The study consisted in spin-echo (SE) T1 sequences (TR 450 ms TE 20 ms) on multiple planes (axial and coronal or sagittal) selected in relation to the site of the tumours into the oral cavity and short-tau-inversion-recovery (STIR) sequences T2 weighted (TR 1800 ms; TE 100 ms; TI 10 ms) acquired on the axial plane. In addiction, for the evaluation of the mandible, SE T1 sequences were acquired on coronal or axial plane with 3-millimetre-thick slices. After administration of gadopentate dimeglumine (Gd-DTPA, Magnevist, Bayern Shering Pharma AG, Berlin, Germany) at 0,2 mmol/kg, T1 fat-suppressed (SPIR) sequences (TR 400 ms;TE 10 ms.) with an acquisition time of 1.43 min on axial planes and SE T1 sequences on multiple planes were used.

MDCT examination was performed using a 4-slice MDCT scanner (Siemens Medical Solutions, Enlargen, Germany). The scans were performed with the patients supine with head first, using the following parameters: slice collimation 4 × 1; tube voltage, 120 kV; effective mAs, 150; slice thickness 1 mm; reconstruction section thickness 1.5 mm; gantry rotation time 0.8 s; field of view (FOV) 35-50 cm.

Unenhanced MDCT images were at first obtained; successively contrast enhanced images were achieved during a late phase after a scan delay of 70s by prior intravenous administration of 110 ml of iodinated non-ionic contrast material (Iomeron 300 mg, Bracco Spa, Milan Italy) at a flow rate of 3 ml/s.

Row data were reconstructed with both soft-tissue and bone algorithms and MDCT-reformatted images in axial, coronal and sagittal planes were obtained.

### Image Analysis

Images were analysed on a workstation commercially available which allows analysis of both MRI and MDCT images.

MDCT diagnostic criteria used for the evaluation of the mandibular bone invasion were:

(i) demonstration of cortical bone defects adjacent to the tumour, in order to determinate the cortical invasion, (ii) evidence of trabecular disruption continuous to the cortical bone erosion, in order to determinate the marrow involvement and (iii) MDCT infiltration signs of the inferior alveolar canal.

MRI diagnostic criteria used for the evaluation of the infiltration of mandibular bone were: (i) the lack of the typical hypointense signal of cortical bone on T1 and T2 replaced by the signal intensity of the tumour, useful to determinate the cortical invasion, (ii) the evidence of hypointense signal on T1, hyperintense on T2/fat-suppressed T2 and the presence of enhancement after Gd-DTPA administration, in order to demonstrate the marrow involvement and (iii) the presence of MRI infiltration signs of the inferior alveolar canal.

By the use of a random number table a radiology research assistant (A.G.), not included in the image analysis, uploaded on the workstation both MRI and MDCT data sets of images; two radiologists (A.V.; M.C.) with respectively 15 and 20 years of experience in head and neck radiology, who missknown the histological results, evaluated in consensus all images indicating the evidence of either marrow or cortical mandibular involvement if present.

Imaging results and findings in agreement to our diagnostic criteria were achieved for each set of MRI and MDCT images by the research assistant not involved in the analysis.

A correlation with the recovered histopathologic results was performed by the research assistant and the pathologist. To determine the reasons for any diagnostic errors, the two readers in consensus retrospectively reviewed both false- negative and false-positive findings at MRI and MDCT images.

### Statistical analysis

MRI imaging and MDCT findings were correlated with histopathologic results.

Sensitivity, specificity, positive predictive value (PPV) and negative predictive value (NPV) of MRI and MDCT were assessed.

McNemar test was used to evaluate the overall accuracy of both imaging techniques in the evaluation of the mandible involvement by the SCC.

Differences in the accuracy, sensitivity, specificity, PPV and NPV were calculated at a statistical significance of P < .05.

Statistical analysis was performed with the SPSS 13.0 statistical packadge (SPSS, Chicago, IL, USA).

## Results

At pathological examination, evidence of mandibular invasion was demonstrated in 14 (39%) patients while no bone invasion was present in 22 (61%) patients. Examining the mandibular involvement three main patterns of the infiltration were highlighted: (i) transcortical spread with marrow involvement, (n = 9), (ii) marrow infiltration by alveolar ridge without cortical erosion in patients edentolous (n = 3) and (iii) periosteal infiltration (n = 2). The sensitivity, the specificity, the accuracy, PPV and NPV of MRI and MDCT in the assessment of mandibular involvement are reported in table 2.

**Table 2:** Sensitivity, specificity, accuracy, predictive positive value (PPV), negative predictive value (NPV) of MDCT and MRI in the evaluation of mandibular involvement

	MDCT	MRI
**Sensitivity**	79% [11/14]	93% [13/14]
**Specificity**	82% [18/22]	82% [18/22]
**Accuracy**	81,0% [29/36]	86% [31/36]
**PPV**	73% [11/15]	76% [13/17]
**NPV**	86% [18/21]	95% [18/19]

Note. In the blanket parenthesis are presents the numbers used for the percentuals

Percentages may not total 100 because of rounding. The differences between MDCT and MRI were not statistically significant (p > .05)

**Figure 1:**
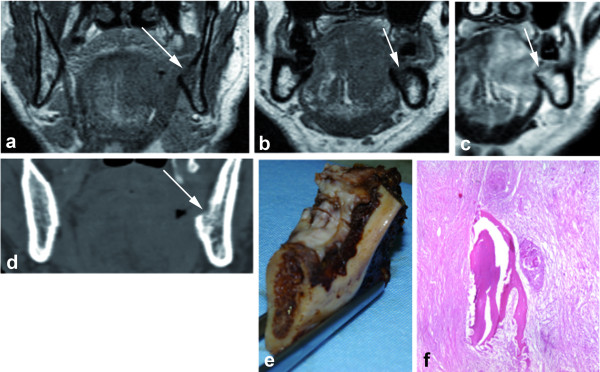
**MRI SE T1 coronal plane (a), SE T1 coronal plane without (b) and after gadolinium (c)**. MRI shows a left floor of the mouth tumour that invading the mandible with cortical erosion and medullary bone involvement (arrows). CT in coronal plane (d) shows cortical invasion (arrow). Gross speciment (e) and histologycal data (f) confirm the cortical and medullary bone invasion (pathological stage pT4).

**Figure 2:**
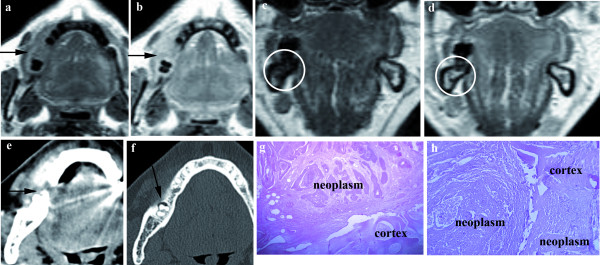
**MRI SE T1 axial planes before (a) and after gadolinium infusion (b); SE T1 coronal planes before (c) and after gadolinium infusion (d)**. MRI shows alveolar ridge carcinoma (arrows) with an infiltration of the cortical and medullary bone (circles). CT in axial planes (e-f) shows an infiltration of the cortex (arrows). Histologycal data (g-h) shows the only cortical bone infiltration.

**Figure 3:**
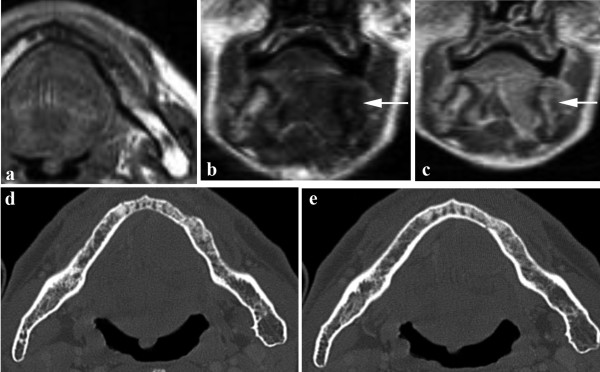
**MRI SE T1 axial (a) and coronal planes before (b) and after gadolinium infusion (c)**. MRI shows a left floor of the mouth tumour with an infiltration of medullary bone, that demonstrates hypointense signal in T1 and enhancement after gadolinium infusion in the edentulous site (arrows). CT in axial (d-e) planes shows normal mandibular cortex. On the histologycal data the mandible was infiltrated (pathological stage T4).

Complessively, MRI showed a trend to have an higher sensitivity compare to MDCT although none statistically significant difference was noted for either sensitivity or specificity (p > .05) (Figure 1, Figure 2, Figure 3). Furthermore, McNemar test didn-t show any difference in the diagnostic accuracy for the evaluation of mandibular invasion between the two modalities (p > .05).

**Figure 4:**
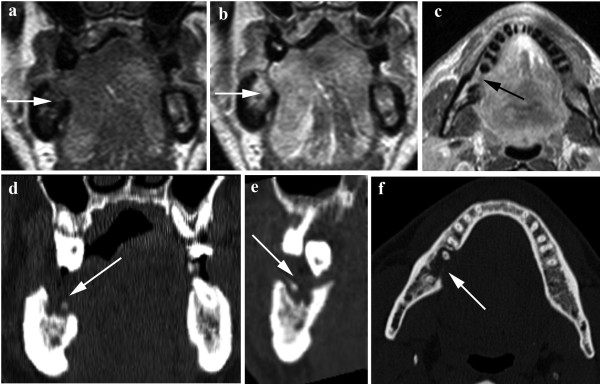
**MRI SE T1 coronal planes before (a) and after gadolinium infusion (b); SE T1 axial plane after gadolinium infusion (c)**. MRI shows a right floor of the mouth tumour with a suspected infiltration of medullary bone in the edentulous site (arrows).  CT in coronal (d) sagittal (e) and axial (f) planes shows a suspected infiltration of the cortex (arrows). The histological result indicated that the mandible was free from neoplastic invasion (pathological stage T3).

On MRI imaging 4 cases were not confirmed at histological examination and they resulted in four false positives (Figure 4), either because of the supposed marrow infiltration (n = 3) or the supposed cortical erosion (n = 1). In one case MRI analysis didn't demonstrate a small cortical erosion (3 mm) and this is resulted in a false negative case at MRI.

At MDCT there were 4 false positives because of the supposed cortical infiltration (n = 3) and the supposed cortical erosion with marrow involvement (n = 1) by the readers.

Three false negatives were reported at MDCT analysis; in 2 cases the infiltration of the marrow by alveolar ridge without a cortical erosion was not reported at MDCT and in 1 case a small cortical erosion (3 mm) was not seen.

## Discussion

Mandibular involvement represents an important issue for preoperative counselling and operative planning since the resection requires the reconstructive surgery with simply metal plate for small later defects or the use of vascularised bone grafts, in the form of free tissue, in those cases in which segmental mandibular resection is performed.

When there is no imaging evidence of mandibular invasion and the tumour is easily separable during surgery from the intact cortical plate, the surgeon may decide to remove the tumour and completely spares the mandible; on the other hand, if the tumour is fixed to the cortical plate and it is not easily separable, the surgeon may perform a marginal (i.e. rim, shave) mandibulectomy, which entails resecting the cortical plate of bone adjacent to the tumour. Instead when there is evidence of bone invasion the standard procedure is represented by the segmental mandibulectomy.

To date, three patterns of mandibular invasion, by squamous carcinoma has been distinguished: the most common is the erosive pattern, characterized by well-defined U-shaped excavation of the mandibular cortex with/without an involvement of the medullary bone, which radiologically appears as a well-defined radiolucent lesions without spicules bone; a second pattern is represented by the effects due to an infiltrative mass which radiologically appears as an ill-defined and irregular lesion [13,14]. Finally, another, more unusual pattern of the mandible's invasion is characterized by neoplastic vascular embolization with cortical integrity [15].

Squamous cell carcinoma spreads along the surface mucosa and the submucosal soft tissue until it approaches ginigival where the tumour may come into contact with the mandible's periosteoum.

The dental sockets represent the mandible's entry way in dentate patients; the tumour cells migrate into the occlusal surface of the alveolus in the edentulous patients and enter the mandible via dental pits [15-17].

Panoramic X-ray (OPG) [18], CT scans, MRI and CT-PET [19,20] represent the imaging techniques for early assessment of the mandibular invasion. OPG efficacy in evidencing early mandibular invasion ranges between 60% and 64%, suffering from an high rate of false negative results [18]. MDCT scans with Dentascan may offer an excellent technique for the evaluation of bone erosion from squamous cell carcinoma with a sensitivity of 95% and specificity of 79%, as reported in a recent work [18]. On the other hand, MRI is generally considered superior to MDCT in the evaluation of the medullary bone space invasion.

However, the diagnostic accuracy of MDCT and MRI in detecting mandibular invasion varies widely, depending on the researchers [5,7,21]. Our results showed higher sensitivity of MRI compared to MDCT although any statistically significant difference was reported probably because of our small study population. In accordance to us, Van den Brekel et al. [12] assessed mandible's invasion on 29 patients and found that MRI compare to MDCT had the higher sensitivity (94%), but lower specificity (73%).

A previous study on the evaluation of the tongue and floor-of-the-mouth tumours by Crecco et al. [6] reported an accuracy of MRI in the evaluation of the mandibular invasion of 93%, while recently, Bolzoni et al. [15] found a higher sensitivity, specificity and accuracy using MRI (93%); the predictive positive value was 87,5% and the negative value was 96%.

Our results are also not completely in accordance with those of Imaizumi et al. [21]; in fact, although they reported similar MDCT results and similar MRI sensitivity, they showed a lower specificity of MRI either for mandibular cortical invasion (54%) or the inferior alveolar canal involvement (70%); these authors gave a presumable explanation of their results that could be influenced by chemical shift artifacts. In our study we had no evidence of chemical shift artifacts that could mimic a mandibular invasion.

Instead, we are more in agreement with the study of Wiener et al. [4] where MRI was superior to MDCT either in the sensitivity or in accuracy while MDCT showed similar specificity compare to MRI.

Furthermore, in our study MRI reported an higher predictive negative value compared to MDCT, while the positive predictive value was similar. However, MRI yielded false-positive cases in the evaluation of the medullary bone invasion.

We used the replacement of the high-signal intensity of the bone marrow on T1 sequences (hypointensity on T1 of the tumour) and contrast enhancement to identify the neoplastic infiltration. This aspect is similar to that create by infiammatory change due to odontogenic disease as dental caries and periodontal disease that shows hypointense signal intensity on T1 and hypeintense in T2 sequences and contrast enhancement; this condition can determine the false positive cases. In our study we reported four cases of false positive at MRI in the evaluation of the marrow involvement; these cases were attributed to a severe periodontal disease or to infiammatory changes due to tooth extraction. In true positive cases when marrow appeared infiltrated, MRI resulted superior to MDCT, particularly in edentolous patients, with infiltration beyond the alveolar ridge without evidence of cortical erosion.

In our study, in one case the abnormal hypointensity on either T1 or T2 of marrow close to the tumour was correctly interpretated as bone sclerosis.

In the evaluation of the mandibular cortical invasion we found one false positive case with MRI and CT, in relation to focal infiltration (< 3 mm.); while in one false positive case with MRI, dental CT- reformatted images was useful to exclude cortical invasion suspected by MRI.

Our study have several potential limitations that merit considerations. First, the methodological limitations inherent the retrospective design of the study, thus our results need to be confirmed in larger prospective studies. Second, our examinations were conducted with conventional MRI image and we are in accordance with Imaizumi et al. that high-resolution images might show further details of the mandible and improve the diagnostic accuracy of MR imaging [21,22]. However, although several investigations has reported the usefulness of high-resolution MRI with small-diameter surface coils in diagnosis of the head and neck region since the differences of spatial resolution between MRI and MDCT still represent a limitation, MR imaging have still shown to have lower spatial resolution than 1-mm-thick CT. Additionally, although we think that could be useful extract quantitative data from both MRI and MDCT images about the deep of the infiltration and the grade of a possible erosion of the mandible, a quantitative image analysis was not performed. Finally, whereas in our study the image analysis was conducted in consensus by two radiologist during one reading session the reproducibily of the study must be verified by further studies which include different indipendent reading sessions.

## Conclusions

In conclusion, the current study suggest that MRI could offer additional value in the evaluation of the relationship between the tumour and the mandible having higher sensitivity and negative predictive value although we reported none statistically significant differences and a similar diagnostic accuracy between MRI and MDCT; we believe that further investigations with high resolution MRI and larger study population should be performed because of the importance of the correct SCC staging at imaging for either surgical or treatment management.

## Competing interests

The authors declare that they have no competing interests.

## Authors' contributions

AV gave a substantial contribution on the study conceptions, participated in the sequence alignment, drafted the manuscript and participated to the qualitative image analysis. AG drafted the manuscript, revised it critically and helped in the analysis. RP participated in the study design and carried out the chart review for the acquisition of the data. VM participated in the design of the study and partecipated to the interpretation of the data. RC performed the chart review of the histopathological data and performed their interpretation. OM performed the literature research and contributed to draft the manuscript. IG performed the statistical analysis. SDG participated to perform the statistical analysis and contributed to the acquisition of the data. GS participated in the study design and revised it critically. MC conceived of the study, participated in its design and coordination and participated to the qualitative analysis. All authors read and approved the final manuscript.
